# Influence of Traditional Sporting Games on the Development of Creative Skills in Team Sports. The Case of Football

**DOI:** 10.3389/fpsyg.2020.611803

**Published:** 2020-12-23

**Authors:** Alexandre Oboeuf, Sylvain Hanneton, Joséphine Buffet, Corinne Fantoni, Lazhar Labiadh

**Affiliations:** URP 3625, Institut des Sciences du Sport et de la Santé de Paris (I3SP), Université de Paris, Paris, France

**Keywords:** motor creativity, fluidity, flexibility, praxical communication, praxeme, traditional sporting game, football (soccer)

## Abstract

The aim of this present study is to investigate the influence of three learning contexts on the development of motor creativity of young footballers (8–9 years old). In team sport, creativity is a fundamental issue because it allows players to adapt in an environment of high social uncertainty. To carry out this work, we suggest a method for assessing motor creativity into ecological situations based on the analysis of praxical communications. Creativity originates from an interaction between divergence and convergence. In our case, the number of communications (fluidity) and the diversity of updated communications (flexibility) are our divergence indicators. Convergence, understood as the ability to make good decisions, is assessed by two expert judges (*R* > 0.90). Sixty boys’ football players (*M* = 8.67; SD = 0.3) coming from three football clubs participated in this research. The study lasted 2 years. Each year, a team of 10 players from each club participated in the research twice a week for 32 weeks (8 months), these groups attended different training sessions: (a) the control group (*n* = 20) followed a classical learning; (b) the decoding group (*n* = 20) attended training focused on learning the praxemes of football; (c) the traditional sporting games group (*n* = 20) followed a training session that was jointly focused on praxemes and the practice of traditional sporting games. The motor creativity of players and groups was assessed both at the beginning and at the end of the year during football matches. Compared to the control group, in the post-test, the group with the highest fluidity is the decoding group (*p* < 0.001) and the one with the highest fluidity is the traditional sporting games group. The latter group is also the one with the best convergence (*p* < 0.001). The results showed that traditional games can help develop players’ creative abilities. This research invites us to investigate the complementarity between the different offered training.

## Introduction

Creativity refers to the ability to generate new, original work that is meaningful in its context (Amabile, 1996; Runco and Jaeger, 2012; Anderson et al., 2014). Creativity is not limited to the fields of “arts and sciences” or great discoveries (Lubart et al., 2015). It is now accepted that creativity can be found in all sectors: graphic, verbal, literary, social, scientific, mathematical, musical and also in the field of games and sports (Parlebas, 1999; Memmert et al., 2013; Oboeuf et al., 2019). Furthermore, there are different levels of creativity (Beghetto and Kaufman, 2007). A thought or action may already have been performed by other people while being original, authentic and meaningful to its author in his own life context. This is the case, for example, when a team sports player manages to make a difficult technical gesture for the first time. However, contribution like Fosbury’s flop or Panenka’s shot can benefit from large-scale recognition by both experts in the field and in the public, helping to make a lasting change in the way we think or act. So there is a continuum that goes from personal creativity (Mini-C), creativity recognized by our different groups of belonging, from our friends (Little-C) or the actors in our fields of expertise (Pro-C), to creative eminence (Big-C).

Irrespective of the field and type of contribution, creative work involves a sequence of thoughts and/or actions called “creative process” (Guilford, 1950, 1967; Lau et al., 2013). Creative act is not spontaneous. It requires cognitive operations that are sometimes unconscious (Cropley, 2006; Vries and Lubart, 2019). Indeed, it is now accepted that creative ideas or actions result from the interaction between divergent thinking (fluency, flexibility, and originality) and convergent thinking (Chermahini and Hommel, 2010; Jaarsveld et al., 2012; Zhu et al., 2019). The diverging phase consists of generating a large number of ideas or behaviors in a given situation (fluency) but also with flexibility, that is, being able to diversify the categories of responses for the same situation (Forthmann et al., 2017, 2019). Originality can be a complementary observation criterion. It allows to focus on the unusualness and statistical rareness of the proposed solutions (Memmert et al., 2013).

The converging phase corresponds, for example, to make the best possible decision in a given situation (Jung and Chang, 2017). It is a global, cyclical process and also a “creative interaction” (Groborz and Necka, 2003). In a particular context, participants or groups that produce the most ideas or actions are often those that take the most adapted and original decisions (Lubart et al., 2013). Creativity has long been thought to be principally associated with certain personality traits (Eysenck, 1993; Furnham and Crump, 2014; Zhang et al., 2018), but it is henceforward known to depend largely on contextual factors (Parlebas, 1999; Memmert et al., 2013; Santos et al., 2016). For example, several studies using the sequential priming paradigm have shown that creativity in a situation increases with the previous environmental stimulations to which the subject has been exposed (Cai et al., 2009; Sassenberg et al., 2017). Bargh (2014) describes priming as part of the process by which sensation is turned into perception. In the field of sport, some studies show that social priming has a positive influence on creativity. If football players are presented with the performance of a creative player, they will tend to have better results on creativity tests (fluency, flexibility and originality) than if presented with the performances of a non-creative player (Memmert et al., 2013; Furley and Memmert, 2018).

In team sports, creativity plays a central, crucial role. Indeed, players frequently adapt themselves to the task constraints by interpreting the behaviors of their partners and opponents (Parlebas, 1999; Furley and Memmert, 2015). To be efficient, players must be unpredictable and adopt new ways to individually and collectively spoil the projects of the opponents (Oboeuf et al., 2009; Furley and Memmert, 2018). However, to our knowledge, there is little or no research dealing with in creativity in an ecological situation (Oboeuf et al., 2019). This is probably due to the difficulty of finding reliable indicators because motor behaviors, unlike verbal activities, occur in the three dimensions of space (Birdwhistell, 1970; Parlebas, 1999; Oboeuf, 2010).

Creativity can be observed in ecological situations (Pic et al., 2018, 2020). The “motor situation” is described like “a set of objective and subjective elements that characterize the motor action of one or more players in a given physical environment when performing a motor task” (Parlebas, 1999, p. 337). In motor games, the motor situation brings together the context and its achievements *in situ*. Creativity is therefore dependent on the context, i.e., the structure of communications to which players are subjected by the rules of the game. We don’t communicate in the same way during a football match or a *balle assise* game (Parlebas, 1999; Collard, 2004; Pic et al., 2019). The “framework” (Goffman, 1974) channels motor behavior and helps to understand the strategic choices of players (Pic et al., 2020). The creative player is the one who, in this bundle of constraints, will manage to be unpredictable for the opponent in his communication choices. Football belongs to the same domain as many other team sports and traditional games in which the players must make decisions under strict conditions of social uncertainty (Márquéz Jiménez and Martínez-Santos, 2014). Every player’s conduct is always communicative (Parlebas, 1999). The player must have the ability to “read the game,” to attribute the right strategic meanings of the motor behavior of other players.

In team sports, praxical communication is divided into two broad interdependent categories (Parlebas, 1999; Dugas, 2005). The first one, is considered a direct communication (DC). It is often the only one of worthy interest, because it is closely related to the achievement of the motor task: it involves either a direct relationship to the object (pass, shot, interception, tackle) or to the body of the partner or opponent (contact). This is the first-degree of interpretation of the players’ behavior. In football, this direct communication accounts for 15–25% of communication (Oboeuf et al., 2009). The second category concerns the signs (or praxemes) that are used as support for these direct communications and ensure the overall dynamics of the game. These praxemes are considered as indirect communication (IC). In football, the “ball’s call” is a sign: if a player produces a run to request a pass, the “running” behavior will mean it while the message. In other words, the signified will be the request for a pass from the partner. Ball’s call, but also holding run, recolocation or body feint, are some of these motor signs (Márquéz Jiménez and Martínez-Santos, 2014). The analysis of these signs is a semiology of the motricity, that corresponds to the semiotricity (Parlebas, 1999; Martínez-Santos, 2007; Ghannouchi et al., 2019; Martínez-Santos et al., 2020). It accounts for 75–85% of communication in team sports (Oboeuf et al., 2009). They are inseparable from the game action.

**FIGURE 1:**
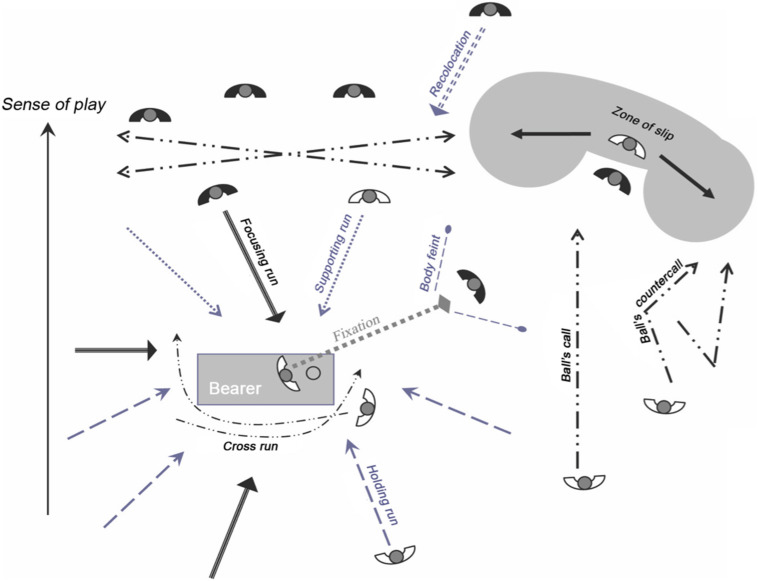
The main praxemes mobilized by footballers during the course of the game. Four praxemes do not appear on the diagram. (i) “Returning run,” which is the driving action of a player who returns to his side after his team loses the ball; (ii) “Tracking run,” which is the driving action of a player who returns to his side by following an opposing player after the loss of the ball by his team; (iii) “Shooting feint”; (iv) “Passing feint.”

Praxeme requires an intention to communicate from the part of the partner or adversary. For example, by advancing toward an opponent who has the ball (focusing run), I want to dissuade him from getting closer to my goal or hinder him in his tactical intentions. Each praxeme can be made up of several clues: position of the supports and of the different body segments, orientation of the bust and head, direction changes, acceleration positioning in space or relatively to other protagonists, and so on. It is these observable elements, these motor behaviors, that will combine together to form a praxeme. The index alone has no communicative value (Winkin, 2001). Praxemes are the “common code,” the directory of signs from the rules that players use to communicate in situations. It is possible to identify the praxemes of a team sport because “signs and sign systems are objectifications insofar as they are available beyond the expression of subjective intentions “here and now”” (Berger and Luckmann, 2006, p. 92). Note that each praxeme has variants without changing its meaning (or signified). These variants differ in three directions: intensity, amplitude and speed (Birdwhistell, 1970; Winkin, 2001; Oboeuf, 2010). Below (Table 1 and Figure 1) we present the 5 direct communications and the 14 praxemes recorded in football (Oboeuf et al., 2009, 2019). The reliability of these praxemes was confirmed by means of a generalizability analysis (Márquéz Jiménez and Martínez-Santos, 2014).

**TABLE 1:** The 19 praxical communications (5 direct communications and 14 praxemes) of football associated with their tactical description.

Direct communications	Categorical core
Interception	Action on the ball by the opponent avoiding him from reaching its destination, cutting the pass line or shot.
Tackle	Action when the opposing player is in ball possession through a struggle or fight which aims to steal it.
Contact	Shoulder to shoulder contact between two adversaries who wish to take the ball.
Pass	Technical action that transmitting the ball by one tap enables the relationship between two teammates.
Shot	All aware throw to the goal in order to make goal.
**Indirect communications (praxemes)**	**Categorical core**
Slip	Motor action (MA) in which the player, coming from an opponent’s back or side of and oriented in relation to the bearer, tries to generate free space for himself for the next action.
Ball’s call	MA in which the player anticipates a possible pass making acceleration toward the goal with the intention to speed up the play and/or winning position.
Ball’s countercall	MA in which the player does an abrupt acceleration in another direction than the ball’s call, which is necessarily above, with the intention to get free spaces generated by itself to the next motor action.
Cross run	MA characterized by a player who crosses by the nearest part of his own goal from one side to another of the bearer’s teammate, and whose intention is to destabilize the opposing defense.
Supporting run	MA in which the player in front of the bearer makes a run to provide a pass solution.
Holding run	MA in which the player behind the bearer makes a run to provide a pass solution.
Returning run	Run of a player who just lost the ball and quickly returns to his defensive position to make the progress of the adversaries progress more difficult.
Focusing run	Run of a player into the rival bearer or receiver in order to reduce the maneuver margin of the opposite team.
Tracking run	Run of a player who follows his direct opponent in order to reduce the maneuver margin of the opposite team.
Recolocation	MA characterized by lateral or anterior-posterior movements of the player that tries to be located in a suitable area according to teammates, opponents and the ball.
Fixation	MA characterized by a run oriented to an area defended by two opposing players in order to create a numerical superiority and facilitate the pass to a teammate in a generated free space.
Passing feint	Bearer’s MA that makes his opponents believe that he is going to do a pass, while his intention is another one (dodge, fixate, shot or pass to a teammate).
Shooting feint	Bearer’s MA that makes his opponents believe that he is going to do a shot while his intention is another one (dodge, fixate, shoot or pass to a teammate).
Body feint	Bearer’s MA that makes his opponents believe he is going to go on one side while finally going on another.
	

Players must adapt to this sign system, “this secret code” (Oboeuf, 2010), by assigning *in situ* the right meanings to the behaviors of other players. Mastering of praxemes and their articulations is a stake to improve motor creativity (Oboeuf et al., 2019). In team sports, motor creativity operationally refers to: (1) a player’s ability to mobilize a large number (fluency) and a great variability (flexibility) of praxemes, that is to show divergence. Furthermore, originality of praxemes is also a criterion of divergence. We do not deal with originality here because originality concerns only some rare praxemes but not the global sign system of team sports; (2) the ability to make the appropriate motor decisions on a game time sequence, that is to show convergence. The creative player is the one who both energizes the game by multiplying a variety of praxemes and makes the best decisions. Each motor action being unique, it is the one that proves most able to surprise, to weaken the balance of opponents by proposing new and adapted answers to the context in which they manifest themselves (Lubart et al., 2015; Oboeuf and Parlebas, 2018).

Based on the above considerations, the proposed study had two objectives:

(1) To propose a method to assess motor creativity (divergence and convergence) in an ecological situation.
(2) To use this methodology, to compare the influence of three learning contexts (focused either on technique, praxemes, or on praxemes and traditional sporting games) on the evolution of the participants’ motor creativity.

In relation to these objectives, two hypotheses are formulated:

(1) Players’ performances in fluidity and flexibility are correlated. In addition, they would be related to convergence performance. In other words, we think that players who use the most praxemes are also the ones who mostly diversify them. They are also the ones who make the most creative overall services from the experts’ point of view.
(2) Training based on learning praxemes and traditional sporting games improves creativity. These games, because of the diversity of their communication structures, can help to develop the decoding abilities of players, their adaptability and ultimately their creativity.

## Materials and Methods

### Study Design

The use of mixed methods allows the study of the scenario of playful specificity due to the relevance of the temporal order of motor events (Arias-Pujol and Anguera, 2017). Mixed methods (Anguera et al., 2018) allow for the full vision of the subject of study, the flexibility of the conceptual framework and the inclusion of new dimensions (Johnson et al., 2007). These methods are suitable for the analysis of motor creativity in its context and complexity. This choice is justified by the work on purely quantitative aspects (number/diversity of praxemes and quality control of data) with the use of qualitative aspects such as the design of an observation grid and the evaluation of convergence by trained judges (Storme and Lubart, 2012). For this, the use of mixed methods was justified by observational methodology, based on the categories of communications (Parlebas, 1999) and the temporal structure of motor actions.

Our observational methodology is a methodological approach adapted to work on the ecological dimension in sport and physical education (Pic et al., 2018). More concretely, a quadrant III observational methodology is applied (Anguera and Hernández-Mendo, 2016). A design was applied that was: (a) nomothetic, as data on different players were recorded; (b) punctual, because the observation were raised in a precise moment, and (c) multidimensional, since different dimensions (criteria) were taken into account (Pic et al., 2018).

### Participants

The number of football players was 60 boys between 8 and 9 years old (*M* = 8.67; SD = 0.3) from three football clubs in the Hauts-de-France region (France). These three clubs were selected because the coaches agreed to participate in the study and to be trained in new coaching methodologies. The clubs were selected according to accessibility and intentionality (Anguera et al., 1995). The study lasted 2 years. Each year, a team of 10 players from each club participated in the research. Twice a week for 32 weeks (8 months), these groups attended different training sessions: (a) The control group (C; *n* = 20) followed a classical learning as recommended by the French Football Federation (FFF); (b) The decoding group (D; *n* = 20) attended training focused on learning the praxemes of football; (c) The traditional sporting games group (TG; *n* = 20) followed a training session that was jointly focused on praxemes and the practice of traditional sporting games. The motor creativity of players and groups was evaluated at the beginning (September) and at the end of the year (June) during football matches. This study was carried out in accordance with the recommendations of *ethics committee* of the University of Paris (France) with written informed consent from all parents or legals tutors of all participants (Declaration of Helsinki).

### Procedure

We recruited 3 football coaches who coach players 8–9 years old (Under 9 category). They have been playing football for at least 10 years and have been coaching for at least 5 years at the time of the study. These three coaches followed the formation and qualification given by the FFF and have the mandatory diplomas to coach in this category. For 2 years, each coach offered his players specific training sessions. Two sessions of 75 min were proposed per week.

The coach of the control group (*n* = 20 over 2 years) did not use any specific training. His players attended a classical learning as recommended by the FFF. This training concerned essentially technical aspects of the game and some tactical contributions. Sessions around ball driving, control, passing, penalty shootout and finally dribbling were preferred. Mostly, during 2/3 of each session, one of these technical aspects was individually addressed with a few motor interactions with partners and opponents. The match time that closed the session (20′). An example of a session is presented in the following table (Table 2).

**TABLE 2:** Description of situations offered to players aged 8–9 during a traditional (technical–tactical) training session on the topic of the pass.

Situations	Descriptions of the proposed situations
Warming up (10′) – Goal: to warm up by progressing in the technical mastery of the object.	Each player trots while driving a ball.
Juggling (10′) – Goal: to progress in the technical mastery of the object.	Each player juggles with his strong foot, then with his weak foot and then with the head.
The bowling game (10′) – Goal: to progress in the accuracy of passes (no opponent).	Players go head-to-head, leaving a distance of 20 meters between them. We position a stud between the two players. In turn, each player must try to touch the plot and score a point if he succeeds. Variation: Reduce or increase distances.
Pass and Follow (10′) – Goal: To progress in the accuracy of moving passes (no opponent).	We separate the group into two equal subgroups that we induct in line face to face 10 meters from each other. The first player in Group A drives the ball and passes to the first player in Group B and then moves to the end of Group B., and so on. Variation: Pass made with a touch of the ball.
Passes and follows (combination) then shoots (10′) – Goal: Progress in the accuracy of passes and build a collective action (no opponent).	Five studs are settled out 5 meters apart. Five players position themselves at each plot, listed from A to E. Player A passes to Player B and then takes his place. Player B passes to Player C and then takes his place, and so on. The last player controls and strikes on goal, and so on. Variation: passes made without control.
Match (20′) – Goal: to put players in situations to contextualize the work done on the pass.	
Return to calm (5′) – Goal: stretch and exchange on the theme of training.	

The other two coaches took part in a twenty-hour training course focused on learning praxemes. The decoding group (*n* = 20) only focus on learning and decoding praxemes and therefore, mainly, on playing off the ball. Each session focuses on one of the 14 praxemes presented above (“Ball’s call,” “Holding run,” “Body feint,” and so on.). In each proposed situation, we made sure to always consider the decoding signs in order to facilitate their learning. In other words, in every situation, there was always at least one opponent and/or partner whose motor behaviors might be interpreted. Each situation offers social uncertainty. Each session ended with a match time like for the control group (20′). The traditional sporting games group (*n* = 20) participated also in a training focused on decoding praxemes. But one or two training situations were substituted by the practice of traditional games. The coach in charge of this group knows the traditional sporting games because he holds the Aptitude Patent for The Functions of Animator. He participated in a 5 hours overtime training. Below is an example of a session for this group (Table 3).

**TABLE 3:** Description of the situations offered to players aged 8–9 during a training session to decode the praxemes on the theme of “ball calling.” A traditional game called “4 corners game” also allows you to work on this theme.

Situations	Descriptions of proposed situations
Warming up (10′) – Goal: Learning to spot accelerations.	The players are by 3 and trotted as they please on the field. A player is in possession of the ball. As soon as one of his two partners engages a brief acceleration, the player with the ball transmits to him in the race, and so on.
“4 corners game” (10′) – Goal: to learn how to spot partners’ start-ups to act.	We delineate a square of 5 × 5 meters with 4 studs. Initially, a player is in the middle. The game begins when the other four players hold their corners: the foot must touch the stud. You can only go back to your corner after you’ve occupied another corner or the middle. It’s about leaving your corner and finding another one before the middle person catches us. It’s a game of races and collective combinations.
The 2-on-1 (15′) call – Goal: to stand out thanks to body feints and ball calling.	A space of 20 meters by 10 meters is delimited for three players. A player owns the ball at one end of the field while his partner is marked by an opponent. This partner must surprise his opponent by using body feints and then a brisk acceleration. The ball carrier must pass the ball to him in the race and the player must manage to retrieve it and cross the line to the other end of the 20 meters without being “caught” by his opponent.
The 3-on-2 (15′) ball call – Goal: stand out in the right tempo to receive the ball in good conditions.	Players leave the middle of the field. The carrier is in the center circle. 10 meters from him, on either side of the halfway line, are an opponent and a partner. One of his partners must choose the right time to engage in a ball call and distance himself from his direct opponent. The carrier must make the pass in the right tempo to put one of his partners in ideal condition. He can choose to shoot at goal or switch to a better-placed partner. All players participate at the end of the action, including the initial carrier.
Theme match (20′) – Goal: to put players in a situation to contextualize the work done on the “ball call.” Prohibition of passing the ball to a static player.	
Return to calm (5′) – Goal: stretch and exchange on the theme of training.	

Data were recorded during football tournaments held at the beginning (pre-test in September) and at the end of the school-year (post-test in June). For each club (*n* = 10 players), two teams of five players were randomly created and participated in the tournament. Consequently, each year (n and n + 1), we had six teams of five players who participated in each tournament. Each team played 4 matches for 12-min games. For example, Team 1 of the Control group 1 played against the two teams in the decoding group and the two teams in the traditional sporting games group. Indeed, players from the same club did not play any match together. The teams were the same during the pre-test and post-test. In each of the four tournaments organized during the 2 years (2 in October and 2 in May), each player was observed for 4 matches of 12 or 48 min. This represents 2880 min of play to be analyzed for all players.

Each match was recorded through the use of two cameras so that it would be possible, in case of doubt in the observations, to resort to a second angle of vision. A single recording was made from the beginning to the end of the game. To carry out the analysis of data, 20 students (football specialists) were trained to recognize practices in a gaming situation. This training includes 6 h of theoretical inputs and 10 h of practice. Each player’s direct (*n* = 5) and indirect (*n* = 14) communications were analyzed by two observers to account for their ability to demonstrate fluency and flexibility (divergence). Convergence is an assessment of overall delivery, the ability to make the right decisions. For each player, this was carried out by two trained judges who must assign a score between 1 and 10. A player close to 1 is considered a non-creative: he makes decisions that never surprise his opponents and neither destabilize his partners. The player close to 10 is considered as very creative: he makes decisions that surprise his opponents and help his partners.

### Data Quality

In order to determine the data quality (Márquéz Jiménez and Martínez-Santos, 2014), inter-observer reliability and validity tests were carried out. Once the observers had uploaded the video into the Lince program, they started to record, separately (Gabín et al., 2012). Each time, the observer detects a praxeme or direct communication from the player, he presses the corresponding button to record the information. Pearson and Spearman correlation coefficients were used. Their values always exceeded 0.96, thus indicating a high correlation between the inter-observer measurements. To assess convergence, for each player, two independent raters (soccer experts with longtime coach occupations and high-level trainer certifications) judged global behaviors. They did not know the objectives of the work. The inter-judge reliability coefficient was above the critical limit of 0.80. Indeed, inter-judges correlation coefficients are all greater than 0.90.

### Variables

We first computed the sum of occurrences of respectively direct (DC) and indirect (IC) communications for the PRE and POST conditions. The fluency, flexibility and convergence were also computed for participants belonging to all the groups (decoding D, traditional games TG, and control C).

The fluency (FLU) was defined as the sum of all the direct and indirect communications used by the participant. The flexibility (FLEX) is the number of different communications regardless of the number of occurrences of each communication. The convergence corresponds to the mean score awarded to each participant by the experts during evaluations. We also computed DFLU, DFLEX, DCONV the differences between observed variables values between the beginning and the end of the training.

### Data Analysis

Consequently, we obtained from the score six dependent variables (FLU, FLEX, CONV, DFLU, DFLEX, DCONV) with two factors: GROUP which is a three-level factor identifying the group of practices, and PRE/POST which is a two-level factor. To measure the effect of these factors on the dependent variables, we first processed ANOVAs on FLU, FLEX, CONV with the GROUP and PRE/POST factors, and then ANOVAS on DFLU, DFLEX and DCONV with the GROUP factor only, to question the effect of the type of training on the progressions of children. For this last analysis we also estimated the effect size (η^2^). *Post hoc* tests with Bonferroni corrections were also made to compare the mean values of variables.

The significance threshold was set to 0.05, and a tendency to significance was determined by a *p* value inferior to 0.1 but not inferior to 0.05.

### Materials

Judges recorded the direct and indirect communications with the Lince software (Gabín et al., 2012) and the JASP statistical software^1^ was used for statistical calculation and analysis.

## Results

### Direct and Indirect Communications

**FIGURE 2:**
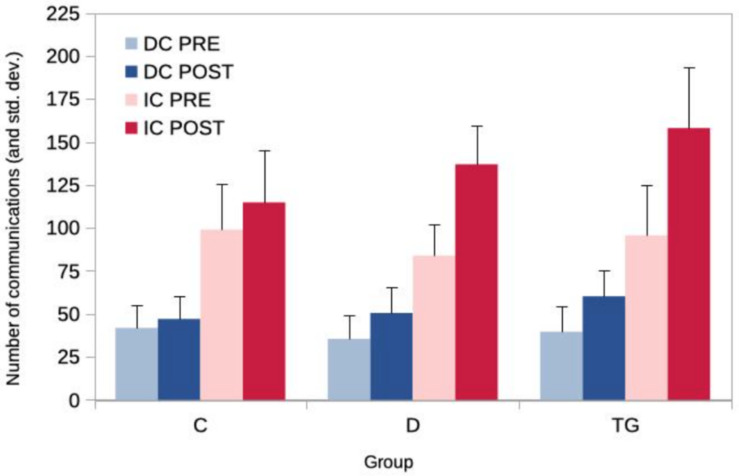
Mean number of direct (DC) and indirect (IC) communications for the PRE and POST measures in the three groups.

The mean amount of direct and indirect communications increased in the three groups. However, this increase seems to be lower in the control group (Table 4 and Figure 2 below).

**TABLE 4:** Means and standard deviations of the number of direct and indirect communications for the PRE and POST measures for the three groups.

	Direct communications		Indirect communications	
GROUP	DC PRE	DC POST	IC PRE	IC POST
C	41,85 (±13,41)	47,1 (±13,13)	99,1 (±26,39)	114,85 (±30,14)
D	35,55 (±13,75)	50,55 (±14,64)	83,95 (±17,84)	137 (±22,71)
TG	39,65 (±14,65)	60,25 (±15)	95,7 (±29,26)	158,05 (±35,21)

The results of ANOVAs are summarized in the Table 5 below. The main factors GROUP and PRE/POST are significant for both DC and IC. However, the influence of the type of training depends significantly on the group since the GROUP × PRE/POST interactions are significant. The progression in the mean amounts of DC and IC is not the same in the different groups since the GROUP factor is significant both for DC and IC.

**TABLE 5:** Results of the ANOVAs for the direct and indirect communications scores, and for the changes in direct and indirect communications between the PRE and POST measures (progression).

	Scores		Progression	
	DC	IC	DC	IC
GROUP	*F*[2,57] = 1.764; *p* < 0.181	*F*[2,57] = 3.23; *p* < 0.047	*F*[2,57] = 6.187: *p* < 0.004; η^2^ = 0.178	*F*[2,57] = 53.5; *p* < 0.001; η^2^ = 0.652
PRE/POST	*F*[l,57] = 57.03; *p* < 0.001	*F*[l,57] = 504.28; *p* < 0.001		
GROUP × PRE/POST	*F*[2,57] = 6.187; *p* < 0.004	*F*[2,57] = 53.5; *p* < 0.001		

*For the DC and IC scores, the effects of the GROUP and PRE/POST factors and their interaction were considered, whereas we obviously analyzed only the effect of the GROUP factor for the progressions.*

*Post hoc* tests show that the mean DC and mean IC do not differ significantly among groups for the PRE measure. For the POST measure, the mean DC number of the TG group exhibits a tendency to differ from the mean DC of the control group (*p*_bon__f_ < 0.061). However, concerning indirect communications, the mean of the TG group differs significantly from the mean of the control group (*p*_bon__f_ < 0.001). This was not the case for the D group.

The mean progression of the TG group for DCs was significantly different from the mean progression of the control group (*p*_bon__f_ < 0.003) whereas the mean of the D group has only a tendency to be different (*p*_bon__f_ < 0.094). For indirect communications, both the TG and D groups exhibit a significant difference with the mean of the control group (*p*_bon__f_ < 0.001).

### Fluency, Flexibility and Convergence

The scores obtained for fluency, flexibility, and convergence increased in the three groups between the PRE and POST measures. Again, the scores of the control group seems to increase less than the other scores (Table 6).

**TABLE 6:** Means and standard deviations for the fluency (FLU), flexibility (FLEX), and convergence (CONV) for the PRE and POST measures in the three groups.

	Fluency		Flexibility		Convergence	
GROUP	PRE	POST	PRE	POST	PRE	POST
c	140,95 (±33,88)	161,95 (±37,10)	12,25 (±1,48)	13,15 (±1,14)	5,6 (±1,35)	5,95 (±1,15)
D	119,5 (±28,03)	187,55 (±31,39)	12,1 (±1,52)	16,15 (±1,57)	4,8 (±0,95)	6,85 (±0,88)
TG	135,35 (±40,31)	218.3 (±44,06)	12,7 (±1,66)	15,7 (±1,89)	5,25 (±1,45)	7.6 (±1,23)

The results of ANOVAs are summarized in the Table 7 below. Concerning the scores for FLU, FLEX, and CONV, the GROUP main factor was not always significant. However, both the PRE/POST factor and GROUP × PRE/POST interactions were shown as systematically significant for the three variables. This means in particular that the effect of the type of training has an influence on the mean progression on participants. This result was confirmed by the ANOVAs made on progressions of children. The GROUP factor had systematically a significant influence on the mean progressions of all the three variables with a moderate to high effect size (η^2^).

**TABLE 7:** Results of the ANOVAs for FLU, FLEX, CONV and for their progressions between the PRE and POST conditions.

	Scores			Progression		
	FLU	FLEX	CONV	DFLU	DFLEX	DCONV
GROUP	*F*[2,57] = 3.351; *p* < 0.042	*F*[2,57] = 8.5; *p* < 0.001	NS	*F*[2.57] = 41.73; *p* < 0.001; η^2^ = 0.594	*F*[2.57] = 17.175; *p* < 0.001; η^2^ = 0.376	*F*[2,57] = 47.28; *p* < 0.001; η^2^ = 0.624
PRE/POST	*F*[1,57] = 393.54; *p* < 0.001	*F*[l,57] = 140.66; *p* < 0.001	*F*[1.57] = 305.66; *p* < 0.001			
GROUP × PRE/POST	*F*[2,57] = 41.73; *p* < 0.001	*F*[2,57] = 17.175; *p* < 0.001	*F*[2.57] = 47.28; *p* < 0.001			

*For the FLU, FLEX, and CONV scores, the effects of the GROUP and PRE/POST factors and their interaction were considered, whereas we obviously analyzed only the effect of the GROUP factor for the progressions.*

**FIGURE 3:**
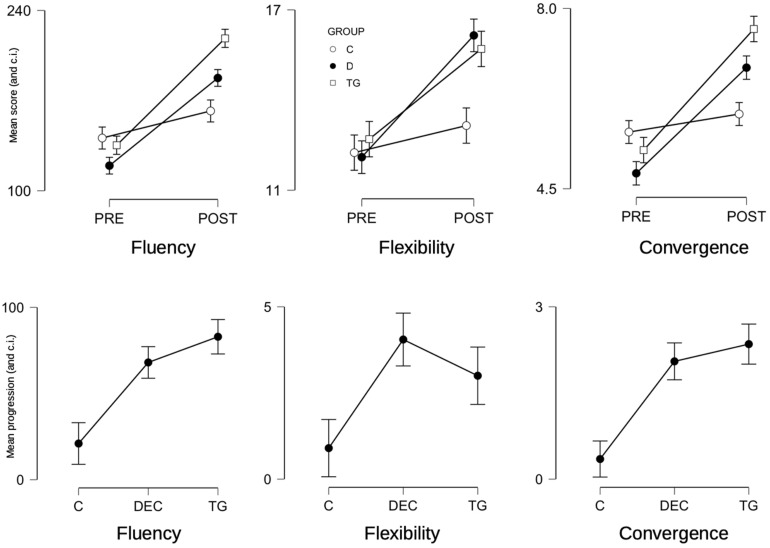
**Top row:** graphs of interactions between the GROUP and PRE/POST factors on the FLU, FLEX, and CONV variables (and confidence intervals). **Bottom row:** effect of the GROUP factor on the progression of the FLU, FLEX, and CONV variables (and confidence intervals) between the PRE and POST measures.

*Post hoc* test with corrections for multiple comparison were used to compare the mean scores and the mean progressions. Results are also displayed of the figures (Figure 3). Concerning fluency, the mean scores of the three groups are not significantly different for the PRE measure. But for the POST condition, the mean fluencies of the TG and control groups are significantly different (*p*_bon__f_ < 0.001). The flexibility means scores of the three groups did not differ in the PRE measure, but mean flexibilities of D and TG groups differ significantly from the mean flexibility of the control group in the POST measure (*p*_bon__f_ < 0.001 for both). Concerning convergence, there was no difference between means of the three groups for the PRE measure, but TG mean convergence differs significantly from the mean convergence of the control group.

The progressions in fluency, flexibility, and convergence of D and TG groups differ significantly from the progression of the control group (*p*_bon__f_ < 0.001). However, there was no significant difference in progression between the D and TG groups.

In conclusion, fluency, flexibility and convergence increased in each group. However, this progression is more important and similar for the TG and D groups.

### Relationship Between Convergence and Both Fluency and Flexibility

In order to investigate the link between scores in fluency and flexibility, and convergence, and their progression, we looked at the correlation between these three variables independently for the PRE and POST measures. The results are summarized in the Table 8 below:

**TABLE 8:** Coefficients of correlation between the FLU, FLEX, and CONV variables in the PRE and POST conditions.

	Scores	
	PRE	POST
FLU × FLEX	*R* = 0.527***	*R* = 0.603***
FLU × CONV	*R* = 0.890***	*R* = 0.893***
FLEX × CONV	*R* = 0.504***	*R* = 0.570***
	Progression	
DFLU × DFLEX	*R* = 0.586***	
DFLU × CONV	*R* = 0.784***	
DFLEX × CONV	*R* = 0.457***	

The three stars (“***”) mark labels a significant correlation (Pearson correlation test) that give a p value inferior to 0.001.

We found that all these variables are significantly correlated. These results confirmed the correlation already demonstrated in the literature between flexibility and fluency (Forthmann et al., 2017). Concerning the scores, the correlation seems to systematically increase in the POST measure. Progressions are also significantly correlated. The effect size of the correlation is important and the highest between fluency and convergence scores (*R*^2^ = 0.8), and fluency and convergence progressions (*R*^2^ = 0.615).

## Discussion

The goal of the present study was to see if contextual factors, different training types here, influenced the motor creativity of young footballers (8–9 years) in ecological situations. In the field of sport, works on social priming have already shown that creativity can increase with environmental stimulations to which the subject has been exposed (Memmert et al., 2013; Furley and Memmert, 2018). The main limitation of social priming studies is that they are mainly carried out in laboratories and not in ecological situations (Doyen et al., 2012; Pashler et al., 2013). However, Bargh (2014) argues that social priming is more likely to have effects on real-world situations than in conventional cognitive tests. Furthermore, few researches, even outside of sport, demonstrated that priming influences creativity in more naturalistic contexts (Berger et al., 2008; Papies et al., 2013). In this study, we proposed a method to assess the influence of the environmental factor on motor creativity in representative settings (Parlebas, 1999; Furley and Memmert, 2018; Pic et al., 2018; Oboeuf et al., 2019; Pic et al., 2020).

Analysis of the rules and repeated observations of the game-playing allowed us to bring out all the communications used by players to be creative, i.e., unpredictable for opponents (Parlebas, 1999; Oboeuf, 2010; Márquéz Jiménez and Martínez-Santos, 2014; Oboeuf et al., 2019). From the recording of communications, we computed two divergence indexes: fluidity and flexibility. The more indirect and direct communications a player uses, the more fluid he or she is considered. The more he/she diversifies them, the more it is considered as flexible. In the literature (Lubart et al., 2015; Forthmann et al., 2017), fluidity and flexibility are always found as strongly correlated (*R* = 0.80 to 0.90). Results from our methodology corroborate those obtained in the literature. The more communications football players use, the more diverse they are. Our coefficients of correlation were *R* = 0.527 (*p* < 0.001) in the pre-test and *R* = 0.603 (*p* < 0.001) in the post-test. Our methodology highlights the same phenomena than in the literature data. We suggest that correlations are weaker because the players have specific tactical roles (right defender, left midfielder, center-forward, etc.) that can sometimes inhibit the use of some communications. For example, a defender will probably make fewer “shooting feints” than an attacker and *vice versa*, the attacker will make fewer “tackles” than a defender. Defenders are often asked to limit risks, which is to value fluidity at the expense of flexibility. On the other hand, attackers are often asked to take risks, which means valuing flexibility.

Another point is worth asking. It concerns the relationship between divergence (fluidity and flexibility) and convergence. We postulated that during a match, a player who diversifies his communications and performs a large number of them, will be considered as more creative by the expert judges (inter-judge correlation: *R* > 0.90). The correlation coefficients between flexibility and convergence were *R* = 0.504 in the pre-test (*p* < 0.001) and *R* = 0.570 in the post-test (*p* < 0.001). The correlation coefficients between fluidity and convergence were even more important: *R* = 0.890 in the pre-test (*p* < 0.001) and *R* = 0.893 in the post-test (*p* < 0.001). Although our results need to be confirmed by other studies, they seem to underline the fact that there is a strong “creative interaction” linking divergence and convergence in the creative process in an ecological situation (Groborz and Necka, 2003; Chermahini and Hommel, 2010; Zhu et al., 2019).

This methodology has been used to investigate the influence of the contextual factors on the development of motor creativity in young footballers aged 8–9 years. What is the learning environment that promotes the best progression in participants?

First of all, it should be noted that, during the pre-test, the average amount of communications (direct and indirect) does not significantly differ between groups. During the pre-test, this is also the case for the indicators of fluidity, flexibility and convergence. This emphasizes the initial homogeneity of the groups and their comparability. On the other hand, for the sample as a whole (*n* = 60), there is a significant difference in the volume of communications used between pre-test and post-test (*p* < 0.001). The interesting result is that this progression depends significantly on the group and therefore on the learning context proposed in the study (*p* < 0.001). The control group is the least creative: it performs the worst for all three indicators (fluidity, flexibility, and convergence). Traditional training seems to be too focused on learning direct communications, i.e., technical gestures (passes, shooting, ball-driving, etc.), to the detriment of indirect communications, i.e., praxemes. However, it is “in the game without the ball that the dynamics of collective duels are based” (Collard, 2004, p. 6). By working the praxemes only with a fortuitous way, coaches cut themselves 75 to 85% of the updated communication during matches (Oboeuf et al., 2009) and this limits the development of motor creativity. There is a “secret code” (Sapir, 1971, p. 46), made up of praxemes and their joints, which is marginalized in this traditional training form.

The results obtained by the Decoding group (D) differ from those in the Control group (C). This group outperformed the control group in the field of fluidity, i.e., in the number of updated communications *in situ*. In particular, there was a strong significant difference for indirect communications (*p* < 0.001). But it should be noted that it is in the area of flexibility that Decoding group is the best performer (Figure 3). The variability in the used praxemes is greater here than for the two other groups (TG and C). Indeed, players used a large part of the available praxemes. The learning of praxemes carried out during training situations is reinvested during matches. This is an indication that an intra-specific learning transfer occurred (Dugas, 2005; Parlebas and Dugas, 2005; Kearney and Judge, 2017). Learning praxemes, i.e., working on semiotricity, is a crucial issue to make the game more dynamic and to develop the motor creativity of participants (Parlebas, 1999). The more players master praxemes, the more accurately they will decode the opposing strategic choices and the more they will be, individually and collectively, creative.

The Traditional sporting Games group (TG) presents also some interesting results. Like the Decoding group, it had the characteristic of being more fluid than the control group, using more significant communications. In fact, this group had the best fluidity. Unlike the Decoding group, these differences also depend on direct communications (*p* < 0.003) and not just indirect communications (*p* < 0.001). In other words, beyond the strong global dynamics associated with praxemes, these players also made more passes, shots and interceptions. Players of the Traditional sporting Games group were also those who performed the best creative performances, in other words, who had the best convergence (Figure 3). As suggested in the literature, there is an interspecific transfer between traditional games and team sports (Dugas, 2005). By being confronted with motivating, complex and varied communication structures (asymmetry and/or imbalance and/or ambivalence and/or instability), players develop their adaptability (Parlebas, 1999; Lavega et al., 2014; Pic et al., 2019). From the point of view of the experts asked to evaluate their performance, they more often surprise their opponents with their motor decisions and ultimately show more motor creativity (Oboeuf et al., 2019). Specifically, if the player “has grasped the interactional system that regulates the participants’ game, he can predict the movements a few moments before their actual occurrence” (Winkin, 2001, p. 120). Our study demonstrates that it is possible to teach the player to better anticipate, to pre-act and to make the right strategic choices in an environment of high social uncertainty (Martínez-Santos, 2007; Ghannouchi et al., 2019).

Among the limitations of this study, it should be noted that the number of participants (*n* = 60) could have been greater. It would also be interesting to replicate this study with other audiences and in other sports to determine if the influence of contextual factors on motor creativity is preserved, increased or reduced. Moreover, the link between the development of motor creativity and the communication structures of the proposed traditional games should be deepened. We also suggested above that the relationship between tactical roles of the game and motor creativity could be more fully asked. On the other hand, the influence, at a given age, of the level of physical, cognitive and emotional development of the participants could also help to better define the specific influence of contextual factors on the motor creativity increase. Finally, to better define the involved mechanisms in the strategic choices made *in situ*, we could conduct interviews of self-confrontations with the players (Aiguier et al., 2015; Martinent et al., 2015). This could bring interesting elements to support the development of motor creativity.

## Conclusion

A methodology for assessing motor creativity in ecological situations has been proposed. Even if its relevance must be confirmed by subsequent studies, the results have highlighted the possibility of designing indicators of divergence and situational convergence. On this basis, we were able to explore the influence of three learning contexts on the motor creativity of players *in situ*. Clearly, the type of proposed training influences the development of creative abilities. The results of this study invite to question the current content of the trainings recommended by the FFF. Learning seems to be too focused on the physical, tactical and technical aspects of the game (Clemente et al., 2016; Gonçalves et al., 2017). These three dimensions offer valuable performance support in group sports, but they can only be efficient if the participant manages to properly decode the behaviors of other players. The challenge is therefore for young footballers to master the “secret code” of their practice. Finally, our study lays the groundwork for a reflection on the complementarity between these different training forms. We think that new proposals should not neglect the necessary creative dynamics that players must mobilize in order to find solutions to the problems they meet.

## Data Availability Statement

The original contributions presented in the study are included in the article/supplementary material, further inquiries can be directed to the corresponding author/s.

## Ethics Statement

The studies involving human participants were reviewed and approved by Ethics Committee–University of Paris. Written informed consent to participate in this study was provided by the participants’ legal guardian/next of kin.

## Author Contributions

AO, SH, and JB have contributed to the theoretical and methodological development of the manuscript. CF and LL have contributed with the data analysis. AO, SH, and LL have prepared results and discussion. All authors contributed to the article and approved the submitted version.

## Conflict of Interest

The authors declare that the research was conducted in the absence of any commercial or financial relationships that could be construed as a potential conflict of interest.

## Footnotes

1 https://jasp-stats.org/
